# The chain-mediating effect of societal pressure on appearance and body image on fear of negative evaluation and social anxiety in women with polycystic ovary syndrome: a cross-lagged panel analysis

**DOI:** 10.3389/fpsyt.2026.1898660

**Published:** 2026-09-28

**Authors:** Li Chen, Qiuyan Liang, Dexin Lv, Lejuan Zeng, Xiangfen Zeng, Huiying Cai, Saiqiong Chen

**Affiliations:** Liuzhou Maternal and Child Health Care Hospital, Liuzhou, China

**Keywords:** body image, chain mediation, cross-lag analysis, fear of negative evaluation, longitudinal studies, physical appearance social pressure, polycystic ovary syndrome, social anxiety

## Abstract

**Objective:**

(Prospective Longitudinal Observational Study) This study aimed to examine the longitudinal chain mediating roles of physical appearance social pressure and body image in the relationship between fear of negative evaluation (FNE) and social anxiety among women with polycystic ovary syndrome (PCOS), and to clarify the temporal sequencing among these variables.

**Methods:**

Convenience sampling was used to recruit 264 women with PCOS. Participants completed the Sociocultural Attitudes Towards Appearance Questionnaire-4 Revised (SATAQ-4R), Body Image States Scale (BISS), Brief Fear of Negative Evaluation Scale (BFNE), and Social Anxiety Scale (SAS) at baseline (T0), 3 months (T1), and 6 months (T2), yielding 242 valid cases. Data were analyzed using measurement invariance testing, cross-lagged panel analysis (CLPA), and bootstrapping.

**Results:**

Full measurement invariance was established across the three timepoints (ΔCFI ≤0.01, ΔTLI ≤0.01). The final CLPA model demonstrated excellent fit (CFI = 0.988, TLI = 0.959, RMSEA = 0.023, 90% CI: 0.021–0.026). T0 FNE positively predicted T1 appearance social pressure (β = 0.25, P <0.001) and T1 SA (β = 0.23, P <0.001), and negatively predicted T1 body image (β = −0.19, P <0.001). T1 appearance social pressure positively predicted T2 SA (β = 0.22, P <0.001). T1 body image negatively predicted T2 SA (β = −0.18, P <0.01). T1 SA positively predicted T2 FNE (β = 0.13, P <0.001). Bootstrap analyses revealed significant indirect effects via T1 appearance social pressure (β = 0.055, 95% CI: 0.028–0.082) and T1 body image (β = 0.034, 95% CI: 0.015–0.053), as well as a significant chain mediation effect (β = 0.018, 95% CI: 0.007–0.029).

**Conclusion:**

Longitudinal evidence supports a chain mediating pathway wherein elevated fear of negative evaluation at baseline is prospectively associated with subsequent increases in appearance social pressure and declines in body image, which in turn correspond to higher levels of social anxiety in women with PCOS. Findings support the potential utility of early psychological screening and targeted interventions addressing maladaptive cognitions and body image disturbances, although causal efficacy requires validation via randomized controlled trials.

## Introduction

In recent years, polycystic ovary syndrome (PCOS) has been a common endocrine and metabolic disorder among women of childbearing age, and its associated psychological and behavioral problems have attracted increasing attention (1). Epidemiological data indicate that PCOS patients not only experience physical symptoms such as menstrual disorders, infertility, hirsutism, acne, and obesity but also have a significantly higher incidence of psychological problems such as anxiety, depression, and social anxiety compared to healthy individuals (2, 3). A large number of studies have confirmed that the quality of life of women with PCOS is generally impaired, and the negative perception of appearance (such as body image disorder and appearance anxiety) is an important source of their psychological stress (4, 5).

However, most existing studies focus on emotional symptoms like anxiety and depression, and there is a lack of discussion on the longitudinal development mechanism of social anxiety. In particular, there is a lack of systematic examination of the dynamic relationship between social pressure related to appearance, body image, and fear of negative evaluation (FNE) (6). FNE is the core of social-anxiety cognitive susceptibility factors. According to the cognitive–behavioral model of social anxiety, excessive worry about others’ negative evaluations can significantly increase an individual’s anxiety level in social situations (7).

For PCOS patients, changes in appearance characteristics such as hirsutism, acne, and obesity make them more vulnerable to negative evaluations from family, peers, and society, which intensifies their attention to appearance and social avoidance behavior (8). Cross-sectional studies have found that FNE is significantly positively correlated with social anxiety, but these results are unable to reveal the causal direction and chronological order between them (9, 10).

As an important psychosocial factor, societal pressure on appearance (SPA) plays a key role in the relationship between body image and mental health in women with PCOS. Sociocultural theory emphasizes that high appearance expectations from the media, peers, and family can be internalized into individual self-requirements, which in turn leads to body dissatisfaction and social avoidance (11). Due to the deviation of appearance characteristics from social aesthetic norms, PCOS patients are more likely to feel the “appearance pressure” from the outside world. This pressure not only directly affects their social confidence but also indirectly exacerbates social anxiety through the mediating effect of FNE (12). However, longitudinal research on the effect of social appearance pressure in the PCOS population is still relatively scarce, particularly in examining its mediating effect between FNE and social anxiety.

Body image is a comprehensive reflection of an individual’s cognitive, emotional, and behavioral tendencies toward their own body. Studies have shown that women with PCOS generally have impaired body image, manifested as dissatisfaction with body appearance, shame, and avoidance behavior. This body image disorder is closely related to symptoms such as social anxiety and depression (13). Some studies have further found that body image is not only related to objective appearance characteristics but also affected by subjective experiences such as social comparison and appearance evaluation (14, 15). Based on the experiential avoidance theory, when individuals are highly dissatisfied with their body image, they will avoid social situations to relieve their inner pain, thereby exacerbating social anxiety (16). These three theories are not mutually exclusive. Specifically, the cognitive–behavioral model establishes FNE as the core cognitive vulnerability, sociocultural theory elucidates the origins and internalization of SPA, and experiential avoidance theory explains the transition from body image disturbance to behavioral avoidance. Together, they constitute the theoretical framework of this study (**Figure 1**). Therefore, body image may play a chain-mediating role between FNE and social anxiety; that is, FNE further damages body image by increasing outward social pressure, ultimately leading to an increase in social anxiety.

**Figure 1:**
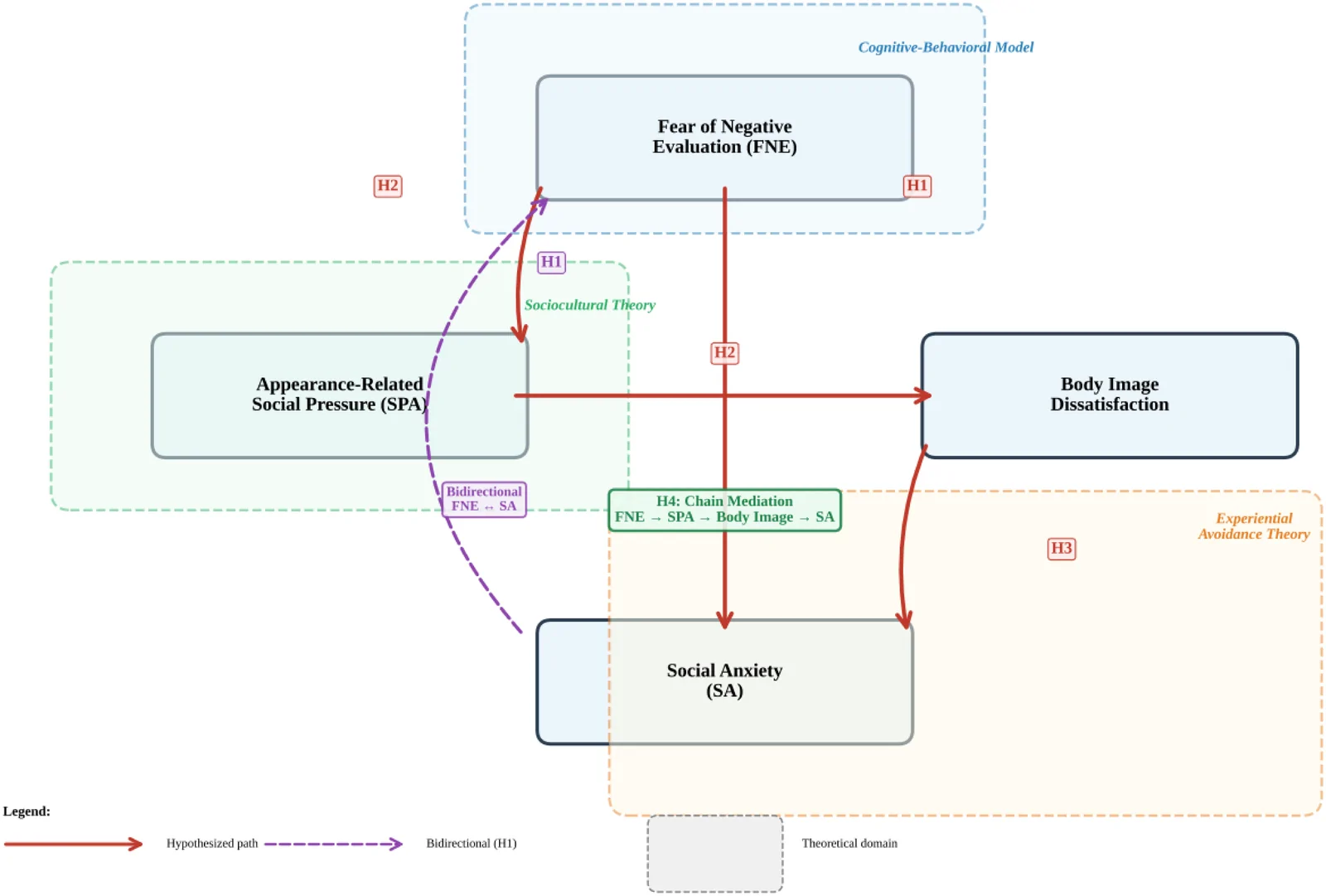
Integrated theoretical framework and hypothesized model.

Studies have found that in teenagers, variables such as obesity, being laughed at, low self-esteem, and FNE can influence social anxiety through a chain path (17, 18), but the validation of a similar mechanism in PCOS patients is still lacking. Moreover, most prior studies have relied on cross-sectional designs, which cannot establish temporal precedence and thus limit causal inference. In contrast, cross-lagged panel analysis (CLPA) addresses these limitations by repeatedly measuring the same participants across multiple timepoints. This approach effectively disentangles autoregressive effects from cross-lagged effects while controlling for baseline levels and measurement error, thereby clarifying temporal–causal relationships. Consequently, CLPA provides an optimal methodological framework for elucidating the longitudinal chain mediation mechanisms linking appearance social pressure, body image, fear of negative evaluation, and social anxiety.

In view of this, the following hypotheses are proposed in this study:

Hypothesis 1 (cross-lag main effect): There is a significant longitudinal bidirectional predictive relationship between FNE and social anxiety in PCOS patients; that is, FNE at T0 can positively predict social anxiety at T1, and vice versa (Theoretical basis: The cognitive–behavioral model of social anxiety; FNE and SA exhibit a mutually reinforcing vicious cycle (10)).
Hypothesis 2 (chain mediation path 1): Social appearance stress mediates the relationship between FNE and social anxiety; that is, higher FNE will enhance perceived social appearance stress, which then induces or aggravates social anxiety (Theoretical basis: Sociocultural theory; elevated fear of negative evaluation heightens sensitivity to appearance-related social cues and amplifies perceived social pressure (11, 19)).
Hypothesis 3 (chain mediating pathway 2): Body image plays a longitudinal mediating role between FNE and social anxiety; that is, FNE damages an individual’s body image (increases dissatisfaction and shame), which then leads to an increase in social anxiety (Theoretical basis: Experiential avoidance theory; body image disturbance precipitates social avoidance behaviors (16, 20)).
Hypothesis 4 (chain mediation model): There is a longitudinal chain-mediating effect of physical appearance social stress and body image in the effect of FNE on social anxiety; that is, the chain conduction path of FNE → physical appearance social stress → body image → social anxiety is established (Theoretical basis: Integrating the cognitive–behavioral, sociocultural, and experiential avoidance frameworks to form a complete cognition–environment–experience–behavior mediational chain).

However, three critical gaps remain in the extant literature. First, while most studies have focused on generalized emotional symptoms such as anxiety and depression, the longitudinal mechanisms underlying social anxiety remain inadequately explored. Second, the dynamic interplay among appearance social pressure, body image, and fear of negative evaluation has yet to be systematically investigated in women with PCOS. Third, the predominance of cross-sectional designs precludes the establishment of temporal sequencing among variables, thereby constraining causal inference. Furthermore, although women with PCOS—characterized by phenotype-specific symptoms such as hirsutism and acne—constitute a high-risk population for social anxiety and body image disturbances, their unique psychopathological mechanisms distinguish them from the general population, necessitating targeted research. To address these gaps, the present study conducted a prospective longitudinal observational design. By tracking levels of fear of negative evaluation, appearance social pressure, body image, and social anxiety across multiple timepoints, we aimed to test the four aforementioned hypotheses, elucidate the chain mediation pathway linking fear of negative evaluation to social anxiety, and provide a robust theoretical and empirical foundation for the early identification and intervention of social anxiety in this vulnerable population.

## Participants and methods

### Participants

From July 2024 to June 2025, convenience sampling was used to select 264 patients with PCOS who were treated in a Class III Grade A hospital in Liuzhou as the survey objects.

Prior to the start of the study, approval was obtained from the medical ethics office of Liuzhou Maternal and Child Health Care Hospital (batch number: [2024] 06035).

Inclusion criteria: According to the China guidelines for PCOS, the standards are as follows: high androgen performance (21); ultrasound showed polycystic ovarian changes; ovulation disorder; complete clinical data; the patient was first diagnosed with PCOS; aged 18 to 40 years old; the patient has good compliance and can actively cooperate with the study.

Exclusion criteria: Comorbid organ dysfunctions (e.g., hepatic or renal impairment); autoimmune deficiencies (e.g., systemic lupus erythematosus); comorbid chronic psychiatric disorders (e.g., major depressive disorder, anxiety disorders, bipolar disorder) with a history of psychiatric treatment within the past 6 months; use of drugs affecting glucose and lipid metabolism or sex hormone levels within the past 3 months; combined with malignant tumors; combined with other endocrine system diseases; complicated with cardiovascular diseases; with infectious diseases such as hepatitis B; blood system diseases such as coagulation disorders.

The present study employed a cross-lagged panel model to test the hypothesized chain mediation effects. Based on the N:q rule of thumb for structural equation modeling and considering the feasibility of clinical recruitment, a minimum sample size of 200 participants was initially determined (22). This figure satisfies the recommended minimum requirement for complex latent variable models. Accounting for the typically high attrition rate in longitudinal designs, we estimated a 15% dropout rate, yielding a required sample size of n = 200/0.85 ≈ 235. *A priori* Monte Carlo simulation was subsequently conducted to empirically validate statistical power. With α set at 0.05 and an anticipated medium effect size (β = 0.20), the simulation demonstrated that a sample of 235 provided an overall model power of 0.92, with power exceeding 0.80 for all core hypothesized paths, thereby fulfilling the requirements for robust statistical inference. Ultimately, 264 patients were enrolled in this study.

### Survey tools

#### Questionnaire for basic information

The questionnaire was self-compiled by the research team and included the patient’s age, occupation, place of residence, marital status, education level, family monthly income, body mass index, and fertility needs.

#### Social pressure on appearance

Schaefer et al.’s stress subscale of The Sociocultural Attitudes Towards Appearance Questionnaire-4 (SATAQ-4R) was used to measure physical appearance social stress (19). The 16-item SATAQ-4R was scored on a 5-point scale, where 1 = “very inconsistent” and 5 = “very consistent.” Scores range from 16 to 80, with higher scores indicating greater perceived physical pressure from the media, family, peers, and significant others. The scale has good reliability in the Chinese population (23). The Cronbach’s α coefficients of the scale in this study were 0.850, 0.924, and 0.910.

#### Body image

The Chinese version of the Body Image States Scale (BISS), developed by Cash et al. and translated by Yang Xiaoying, was used to measure body image (24). The 6-item BISS was scored on a 9-point scale (1 = “very dissatisfied,” 9 = “very satisfied”). The total score ranged from 6 to 54 points, with higher scores indicating a more positive body image. The Cronbach’s α coefficients of the scale in this study were 0.880, 0.796, and 0.894.

#### Brief fear of negative evaluation scale

The revised Brief Fear of Negative Evaluation Scale (BFNE) was developed by Leary. The instrument has a single dimension with 12 items, of which four items are scored in reverse. On a 5-point scale (1 = “very not fearful” and 5 = “very fearful”), total scores range from 12 to 60, with higher scores indicating a higher level of FNE. The Cronbach’s α coefficients of the scale in this study were 0.865, 0.820, and 0.873.

#### Social anxiety scale

The Chinese version of the Social Anxiety Scale was developed by Mattick and Clarke and revised by Dongmei et al. (25). The scale consists of 19 items in one dimension, of which two items are scored in the reverse direction. On a 5-point scale (1 = “very not worried” and 5 = “very worried”), the total scores range from 19 to 95, with higher scores indicating higher levels of social anxiety. The Cronbach’s α coefficients of the scale in this study were 0.830, 0.804, and 0.796.

### Questionnaire recovery and quality control

This study was approved by the hospital Ethics Committee, and all subjects were enrolled after signing an informed consent form. Baseline data were collected at diagnosis (T0), three months after diagnosis (T1), and during the 6-month follow-up (T2). Assessment timepoints were selected to align with standard clinical follow-up schedules for PCOS management in our center, which typically include reevaluation at 3-month intervals to assess treatment response and symptom progression. Prior longitudinal studies in PCOS populations have demonstrated detectable changes in psychological domains (including body image and anxiety symptoms) within 3- to 6-month windows following diagnosis and initiation of clinical care (14, 30). Shorter intervals (<3 months) may fail to capture meaningful psychological adaptation, while longer intervals (>6 months) risk higher attrition; our selected timeframe balances sensitivity to change with feasibility in a clinical sample. During the follow-up period, all participants received standardized PCOS clinical care per current Chinese guidelines (21). Hormonal regulation was prescribed for 218 (90.1%) participants, predominantly combined oral contraceptives (n = 187, 77.3%) or metformin (n = 31, 12.8%), with a mean treatment duration of 5.2 ± 1.1 months. Lifestyle interventions—comprising individualized dietary counseling (caloric restriction and macronutrient balance guidance) and moderate-intensity physical activity recommendations (150 min/week)—were delivered to all participants at baseline and reinforced at each follow-up visit. Researchers systematically documented each participant’s specific treatment regimen (medication type, dosage, frequency) and adherence status (self-reported adherence rated as “good,” “fair,” or “poor”) at T1 and T2. Adherence rates were 86.4% (209/242) at T1 and 82.2% (199/242) at T2. All subsequent statistical analyses adjusted for receipt of hormonal therapy and lifestyle adherence as covariates to minimize potential confounding effects. Researchers systematically documented each participant’s specific treatment regimen and adherence status to facilitate the control of potential confounding effects in subsequent statistical analyses. Through a combination of online questionnaire distribution, telephone interview, and field survey, the trained research staff regularly administered the Appearance Social Pressure Scale, Body Image Scale, FNE Scale-Short Form, and Social Anxiety Scale. A double-blind responsibility system was used for data collection and data entry. That is, one person was responsible for data collection, and the other person checked and entered the data independently to ensure the authenticity and accuracy of the data.

### Statistical methods

Data were analyzed using SPSS 26.0 (IBM Corp., Armonk, NY, USA) and Mplus 8.3 (Muthén & Muthén, Los Angeles, CA, USA). Descriptive statistics were computed for all variables. Categorical variables are presented as frequencies and percentages (%), while continuous variables are expressed as means ± standard deviations (SD).

Pearson correlation analyses were conducted to examine bivariate associations among study variables. Harman’s single-factor test was employed to assess common method bias. Confirmatory factor analysis (CFA) was performed to evaluate the reliability, validity, and measurement model fit of the instruments.

To test the hypothesized chain mediation effects, a cross-lagged panel analysis (CLPA) framework was utilized in Mplus 8.3. Specifically, the indirect pathway “T0 Fear of Negative Evaluation (FNE) → T1 Appearance Social Pressure (SPA) → T1 Body Image → T2 Social Anxiety (SA)” was specified. Bias-corrected non-parametric percentile Bootstrapping with 5,000 resamples was applied to estimate 95% confidence intervals (CIs) for indirect effects. Effects were deemed statistically significant if the 95% CI did not include zero. This approach avoids the limitations of standalone cross-sectional PROCESS models by preserving temporal sequencing. Importantly, Bootstrapping does not rely on the assumption of normality for the sampling distribution of the indirect effect; instead, it empirically estimates CIs through repeated resampling, offering superior accuracy and power compared to traditional Sobel tests.

Measurement invariance across the three timepoints was tested using multi-group CFA in Mplus. Robust Maximum Likelihood estimation (MLR) was adopted to account for non-normality in the data. The CLPA model was specified to disentangle temporal–causal relationships. To isolate the prospective predictive effect of appearance social pressure on body image while controlling for baseline stability, all cross-lagged paths were adjusted for a set of covariates: age, body mass index (BMI), marital status, educational level, fertility desire, and treatment conditions during follow-up (receipt of hormonal regulation therapy and adherence to lifestyle interventions). This covariate adjustment minimized potential confounding and allowed for a rigorous test of the hypothesized pathways.

Model fit was evaluated using established indices: Comparative Fit Index (CFI) >0.900, Tucker–Lewis Index (TLI) >0.900, Root Mean Square Error of Approximation (RMSEA) <0.080, and Standardized Root Mean Square Residual (SRMR) <0.080 indicated acceptable model fit. The statistical significance level was set at α = 0.05 for all tests.

## Results

### Participant characteristics and attrition analysis

Of the 264 questionnaires distributed, 12 participants were lost to follow-up at T1 and 10 at T2, yielding 242 valid responses (effective response rate: 91.67%). Participants ranged in age from 22 to 40 years, with a mean age of 29.33 ± 2.04 years. Additional demographic and clinical characteristics are summarized in **Table 1**.

**Table 1:** General information of the subjects (*n* = 242).

Items	n	Percentage (%)
Age (years)		
<20	21	8.68
20–30	112	46.28
31–40	109	45.04
Career		
Public institution	35	14.46
Professional technicians	64	26.45
Staff/staff	87	35.95
No regular occupation	56	23.14
Place of residence		
Rural	75	30.99
Towns and cities	167	69.01
Marital status		
Married	113	46.69
unmarried	129	53.31
Monthly household income (yuan)		
<5,000	85	35.12
5,000–	106	43.80
10,000–	51	21.07
Education level		
Junior high school and below	77	31.82
High school	80	33.06
College and above	85	35.12
Body mass index		
<18.5	8	3.31
18.5–23.9	55	22.73
≥24	179	73.97
Fertility needs		
Yes	99	40.91
No	143	59.09

Comparative analyses revealed no statistically significant differences between participants who completed all assessments and those lost to follow-up regarding age, body mass index (BMI), or baseline scores of fear of negative evaluation (all P >0.05). These findings suggest that participant attrition was consistent with the missing at random (MAR) assumption. The participant recruitment and screening flowchart is presented in **Figure 2**.

**Figure 2:**
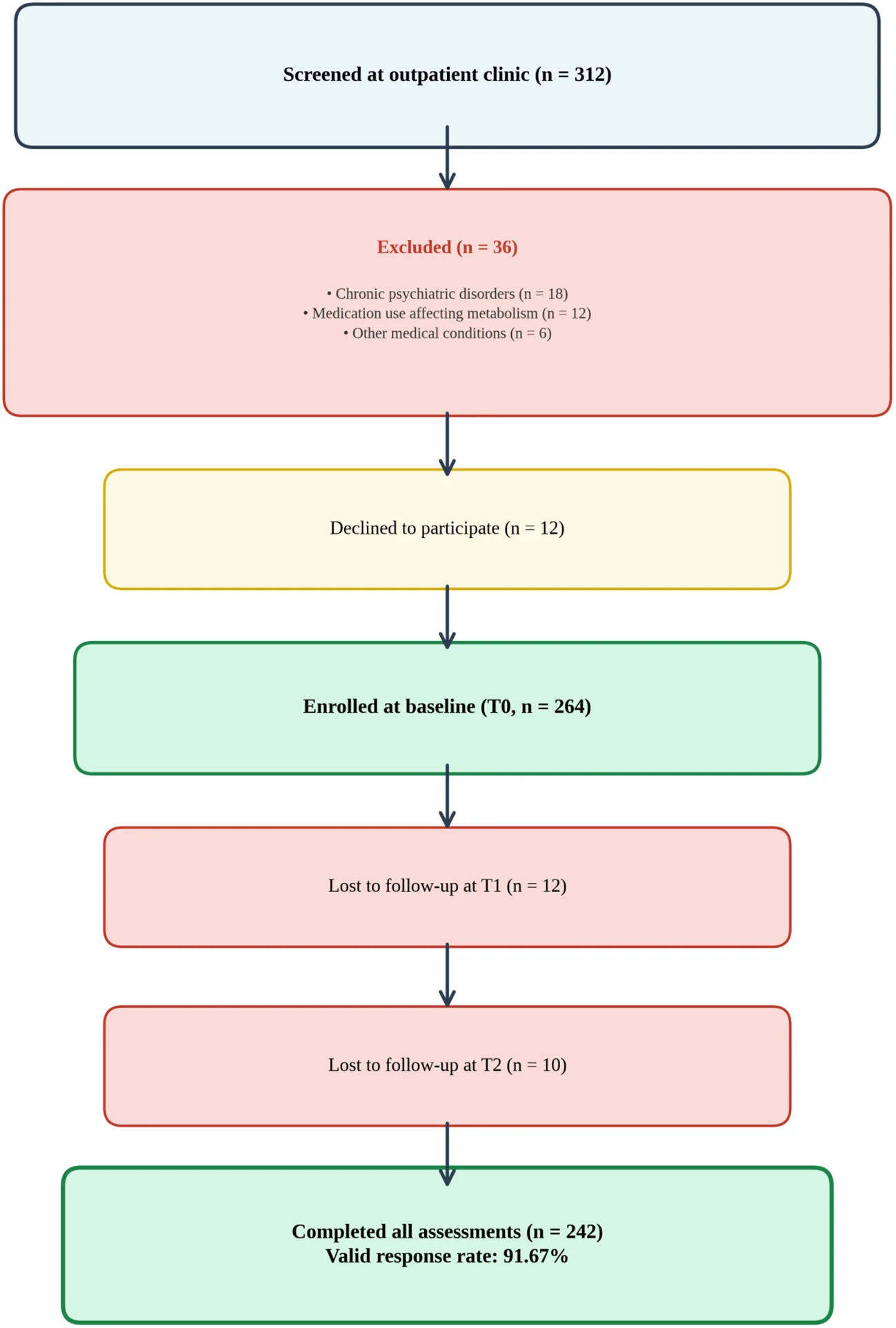
Participant recruitment and screening flowchart.

### Common method bias test

The common method bias test was conducted on the three sets of data using the Harman single-factor test. The variances of the three measurements were 16.24%, 19.37%, and 23.68%, respectively, all of which were less than the 40% critical value (26). Therefore, there is no serious common method bias problem in this study.

### Descriptive statistics and correlation analyses of study variables

Descriptive statistics for all study variables during the 6-month follow-up period following PCOS diagnosis are presented in **Table 2**. Repeated-measures analysis of variance (ANOVA) revealed significant linear trends over time. Scores for appearance social pressure (F = 15.614, P <0.001, η² = 0.061), fear of negative evaluation (F = 23.139, P <0.001, η² = 0.088), and social anxiety (F = 10.133, P <0.001, η² = 0.040) showed significant linear declines, whereas body image scores exhibited a significant linear increase (F = 18.240, P <0.001, η² = 0.070). The effect sizes (η²) indicated that these changes were in the small-to-medium range.

**Table 2:** Scores of physical appearance social stress, body image, fear of negative evaluation, and social anxiety in patients with PCOS (n *=* 242, score, $\bar{\text{x}}$ ± ѕ).

Items	Societal pressure on appearance	Body image	Fear of negative evaluation	Social anxiety
T0	50.24 ± 6.34	35.35 ± 3.92	36.35 ± 3.24	56.24 ± 6.93
T1	48.06 ± 5.95	37.91 ± 3.85	33.93 ± 3.75	54.49 ± 6.86
T2	45.30 ± 5.37	39.89 ± 3.73	31.12 ± 3.58	52.84 ± 6.72
F-score	15.614	18.240	23.139	10.133
P-value	<0.001	<0.001	<0.001	<0.001

Pearson correlation analyses (**Table 3**) demonstrated significant bivariate associations among all variables at each timepoint and across timepoints (all P <0.05). Specifically, fear of negative evaluation, appearance social pressure, and social anxiety were positively intercorrelated, whereas each of these three constructs was negatively correlated with body image. This pattern of correlations is consistent with the prerequisite assumptions for subsequent mediation analyses.

**Table 3:** Correlation coefficient matrix of variables at three time points (*r* value, *n* = 242).

Items	①	②	③	④	⑤	⑥	⑦	⑧	⑨	⑩	⑪	⑫
①SPA T0	1											
②BI T0	−0.405^**^	1										
③FNE T0	0.387^**^	−0.526^**^	1									
④SA T0	0.439^**^	−0.471^**^	0.465^**^	1								
⑤SPA T1	0.528^**^	−0.302^*^	0.371^**^	0.453^**^	1							
⑥BI T1	−0.389^**^	0.467^**^	−0.433^**^	−0.362^*^	−0.382^**^	1						
⑦FNE T1	0.311^*^	−0.425^**^	0.449^**^	0.469^**^	0.369^**^	−0.436^**^	1					
⑧SA T1	0.363^**^	−0.401^**^	0.425^**^	0.464^**^	0.384^**^	−0.442^**^	0.479^**^	1				
⑨SPA T2	0.440^**^	−0.280^*^	0.499^**^	0.315^*^	0.425^**^	−0.479^**^	0.382^**^	0.433^**^	1			
⑩BI T2	−0.295^*^	0.363^**^	−0.314^**^	−0.384^**^	−0.361^**^	0.428^**^	−0.376^**^	−0.456^**^	−0.356^**^	1		
⑪FNE T2	0.277^*^	−0.319^**^	0.401^**^	0.376^**^	0.340^**^	−0.435^**^	0.427^**^	0.429^**^	0.374^**^	−0.395^**^	1	
⑫SA T2	0.312^*^	−0.342^**^	0.378^**^	0.393^**^	0.388^**^	−0.423^**^	0.370^**^	0.403^**^	0.364^**^	−0.426^**^	0.410^**^	1

FNE, Fear of Negative Evaluation; SPA, Appearance Social Pressure; BI, Body Image; SA, Social Anxiety. ^*^ denotes P <0.05, ^**^ denotes P <0.01.

### Reliability and validity of measurement instruments

Confirmatory factor analysis (CFA) demonstrated that all scales exhibited satisfactory model fit at baseline (T0). Specifically, the fit indices for the SATAQ-4R Pressure subscale were χ²/df = 2.14, CFI = 0.962, TLI = 0.954, RMSEA = 0.069, with standardized factor loadings ranging from 0.52 to 0.81. For the Body Image States Scale (BISS), the indices were χ²/df = 1.98, CFI = 0.978, TLI = 0.971, RMSEA = 0.064, and factor loadings ranged from 0.61 to 0.83. The Brief Fear of Negative Evaluation Scale (BFNE) yielded χ²/df = 2.03, CFI = 0.956, TLI = 0.947, RMSEA = 0.066, with factor loadings between 0.48 and 0.79. Finally, the Social Anxiety Scale (SAS) showed good fit: χ²/df = 2.21, CFI = 0.948, TLI = 0.939, RMSEA = 0.071, and factor loadings ranging from 0.45 to 0.77.

Cronbach’s α coefficients for all scales across the three timepoints are reported in the *Measures* section. Collectively, these findings indicate that the measurement instruments possess sound reliability and validity within this PCOS sample, supporting their appropriateness for subsequent structural analyses.

### Measurement invariance testing

Longitudinal measurement invariance was examined using multi-group confirmatory factor analysis (MG-CFA) by sequentially testing three nested models: configural invariance (Model 1; identical factor structure), metric (weak) invariance (Model 2; equal factor loadings across time), and scalar (strong) invariance (Model 3; equal indicator intercepts across time). Model comparisons were evaluated using changes in the Comparative Fit Index (ΔCFI) and Tucker–Lewis Index (ΔTLI), with thresholds of ≤0.010 indicating non-significant deterioration in fit (27).

As presented in **Table 4**, comparisons between Model 1 and Model 2 supported metric invariance for all scales: the SATAQ-4R Pressure subscale (ΔCFI = 0.001, ΔTLI = −0.002), the BISS (ΔCFI = −0.002, ΔTLI = 0.019), the BFNE (ΔCFI = −0.004, ΔTLI = 0.011), and the SAS (ΔCFI = −0.001, ΔTLI = 0.004). Comparisons between Model 2 and Model 3 further supported scalar invariance: the SATAQ-4R (ΔCFI = −0.003, ΔTLI = 0.005), the BISS (ΔCFI = −0.002, ΔTLI = −0.018), the BFNE (ΔCFI = −0.002, ΔTLI = −0.007), and the SAS (ΔCFI = −0.002, ΔTLI = −0.002). All ΔCFI and ΔTLI values were well within the established cutoffs (|ΔCFI| ≤0.010, |ΔTLI| ≤0.010). These findings confirm that the measurement properties of all instruments remained invariant across the three timepoints, thereby supporting the comparability of latent construct scores over time and justifying subsequent cross-lagged panel analyses.

**Table 4:** Equivalence models of physical appearance social stress, body image, fear of negative evaluation, and social anxiety in patients with PCOS.

Variables	Model	*χ^2^*	*df*	CFI	TLI	RMSEA (95%CI)	SRMR
SPA	Model 1	668.510	374	0.945	0.932	0.048 ([0.042,0.052])	0.066
	Model 2	724.672	382	0.946	0.930	0.049 ([0.043,0.053])	0.067
	Model 3	796.525	391	0.943	0.935	0.046 ([0.041,0.054])	0.068
BI	Model 1	960.823	478	0.965	0.950	0.047 ([0.043,0.052])	0.076
	Model 2	972.841	490	0.963	0.969	0.049 ([0.043,0.054])	0.079
	Model 3	994.314	504	0.961	0.951	0.048 ([0.042,0.051])	0.080
FNE	Model 1	142.728	66	0.937	0.925	0.052 ([0.041,0.060])	0.037
	Model 2	158.117	73	0.933	0.936	0.051 ([0.040,0.062])	0.040
	Model 3	166.146	80	0.931	0.929	0.050 ([0.041,0.061])	0.041
SA	Model 1	397.223	158	0.952	0.938	0.058 ([0.051,0.065])	0.047
	Model 2	406.025	166	0.951	0.942	0.056 ([0.051,0.062])	0.048
	Model 3	422.583	176	0.949	0.940	0.056 ([0.049,0.063])	0.050

FNE, Fear of Negative Evaluation; SPA, Appearance Social Pressure; BI, Body Image; SA, Social Anxiety. χ^2^ is chi square, *df* is degrees of freedom, CFI is comparative fit index, TLI is nonbenchmark fit index, RMSEA is root mean square of progressive residuals, SRMR is normalized root mean square residuals.

### Cross-lag analysis of the chain mediating effect of SPA and body image on the relationship between FNE and social anxiety in women with PCOS

#### Model comparison and selection

Four competing models were constructed and evaluated against each other to determine the optimal analytical framework (**Table 5**). These included: a baseline model (M1, autoregressive paths only); a unidirectional predictive model (M2, FNE predicting all other variables); a unidirectional predictive model (M3, SPA and SA predicting all other variables); and an integrated model (M4, incorporating all bidirectional paths).

**Table 5:** Model fit and comparison results.

Models	*χ^2^*	*df*	CFI	TLI	RMSEA	Δ*χ^2^ (df)* vs. M1	Δ*χ^2^ (df)* vs. M4
M1	2,364.225	502	0.936	0.924	0.094	–	–
M2	1,158.137	382	0.973	0.969	0.042	924.256 (182)	522.338 (182)
M3	1,180.330	378	0.967	0.951	0.043	902.114 (180)	562.130 (180)
M4	689.152	202	0.988	0.959	0.023	–	–

M1 is the baseline model, M2 and M3 are one-way prediction models, and M4 is the integrated model, ^*^ which is P <0.01.

Comparative analyses revealed that M1, containing only autoregressive effects, exhibited the poorest fit (RMSEA = 0.094) and was therefore discarded. While M2 (unidirectional FNE effects) and M3 (unidirectional SPA/SA effects) demonstrated improved fit over M1, both were theoretically limited by their assumption of strictly unidirectional causality, which fails to capture the typical reciprocal dynamics inherent in psychological constructs.

In contrast, the integrated model (M4) emerged as the optimal choice. Statistically, M4 outperformed M2 and M3, yielding superior fit indices (CFI = 0.988, TLI = 0.959) and a lower RMSEA value (0.023). Furthermore, the chi-square difference test indicated that M4 fit the data significantly better than M1 (Δχ², P <0.001). Theoretically, M4 aligns with the cognitive–behavioral model of social anxiety, which posits reciprocal influences among core variables. Consequently, M4 provides a more comprehensive representation of the dynamic interplay among psychological symptoms in women with PCOS and was selected as the final model for hypothesis testing.

#### Cross-lagged path coefficients

After adjusting for covariates (age, BMI, marital status, educational level, fertility desire, and treatment conditions during follow-up), the direction and significance of all core cross-lagged paths remained substantively unchanged, indicating the robustness of the findings. The final standardized path coefficients are illustrated in **Figure 3**.

**Figure 3:**
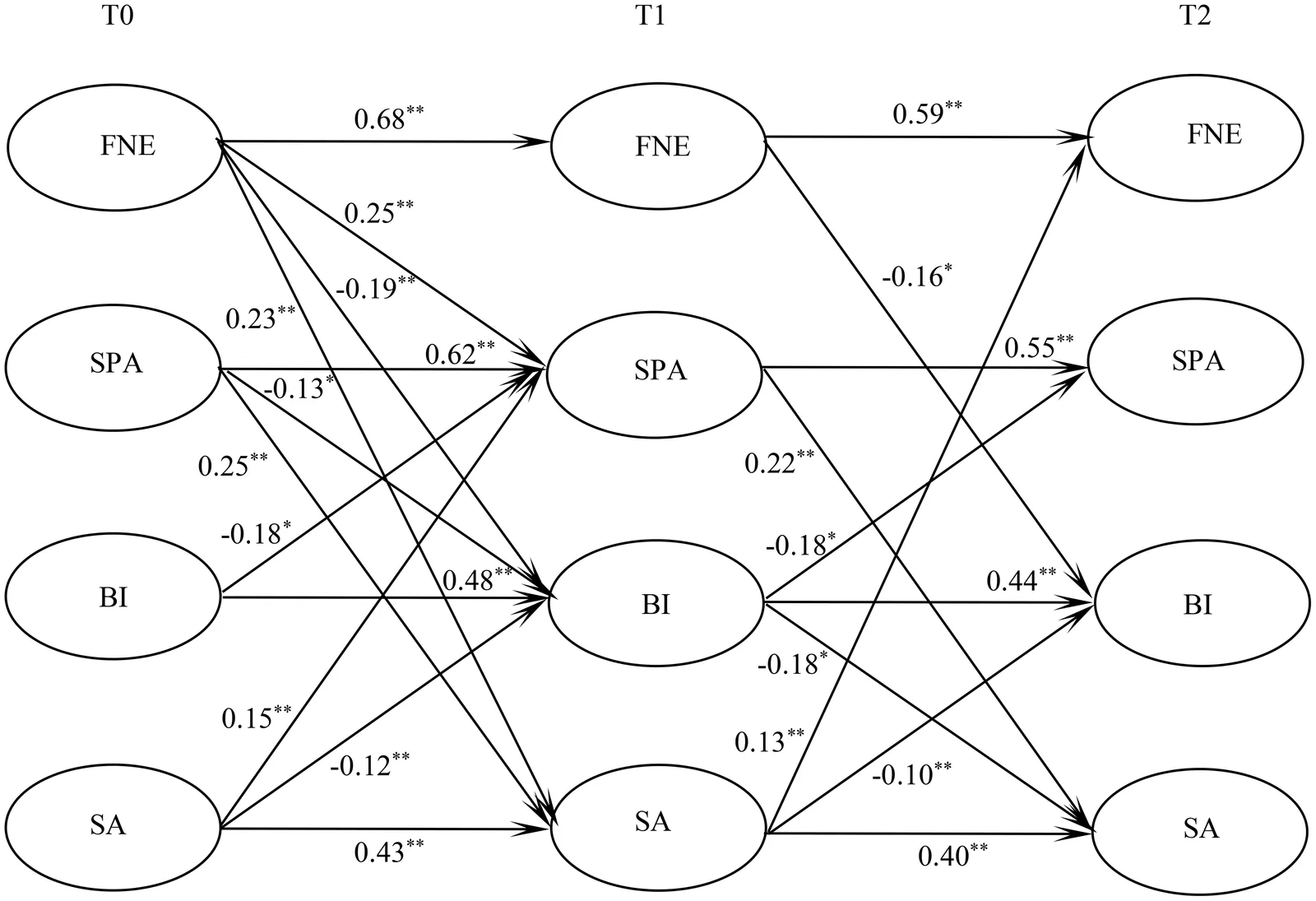
Cross-lagged path diagram of the chain mediating effect of physical appearance social stress and body image on the relationship between fear of negative evaluation and social anxiety in women with PCOS. Note: Only the significant standardized paths are presented. *P <0.01, **P <0.001.

All autoregressive paths were statistically significant (β = 0.42–0.68, all P <0.001), confirming the temporal stability of the constructs over time. Regarding the cross-lagged effects, baseline (T0) fear of negative evaluation (FNE) significantly and positively predicted T1 appearance social pressure (SPA; β = 0.25, P <0.001) and T1 social anxiety (SA; β = 0.23, P <0.001), while negatively predicting T1 body image (β = −0.19, P <0.001). T0 SPA negatively predicted T1 body image (β = −0.13, P <0.01) and positively predicted T1 SA (β = 0.25, P <0.001). Furthermore, T1 FNE negatively predicted T2 body image (β = −0.16, P <0.01), T1 SPA positively predicted T2 SA (β = 0.22, P <0.001), T1 body image negatively predicted T2 SA (β = −0.18, P <0.01), and T1 SA positively predicted T2 FNE (β = 0.13, P <0.001). Details of the path coefficients are presented in **Table 6**.

**Table 6:** Standardized parameter estimates of the final cross−lagged panel model.

Path	T0 → T1 (β)	SE	P	T1 → T2 (β)	SE	P
Autoregressive paths						
FNE → FNE	0.68	0.04	<0.001	0.59	0.05	<0.001
SPA → SPA	0.62	0.04	<0.001	0.55	0.05	<0.001
BI → BI	0.48	0.05	<0.001	0.44	0.05	<0.001
SA → SA	0.43	0.05	<0.001	0.40	0.05	<0.001
Cross-lagged paths						
FNE → Others						
FNE → SPA	0.25	0.04	<0.001	–	–	–
FNE → BI	−0.19	0.04	<0.001	−0.16	0.05	0.003
FNE → SA	0.23	0.05	<0.001	0.10	0.04	0.078
SPA → Others						
SPA → BI	−0.13	0.04	0.002	−0.03	0.05	0.372
SPA → SA	0.25	0.04	<0.001	0.22	0.04	<0.001
SPA → FNE	0.07	0.04	0.124	0.04	0.05	0.289
BI → Others						
BI → FNE	−0.05	0.04	0.210	–	–	–
BI → SA	−0.06	0.04	0.156	−0.18	0.05	0.008
BI → SPA	–	–	–	–	–	–
SA → Others						
SA → FNE	–	–	–	0.13	0.04	<0.001
SA → SPA	–	–	–	–	–	–
SA → BI	–	–	–	–	–	–

FNE, Fear of Negative Evaluation; SPA, Appearance Social Pressure; BI, Body Image; SA, Social Anxiety. All estimates are standardized. p <0.05, p <0.01, *p <0.001. Covariates (age, BMI, marital status, educational level, fertility desire, and treatment conditions) were controlled for in the model. Dashes (–) indicate paths not estimated in the final model. Non-significant paths are reported without asterisks.

#### Chain mediation effects

Bias-corrected bootstrap analyses with 5,000 resamples were conducted to decompose the total effect. The results indicated that the direct effect of T0 fear of negative evaluation (FNE) on T2 social anxiety (SA) was not statistically significant (β = 0.07, 95% CI [−0.02, 0.16], P >0.05). However, significant indirect effects were observed. Specifically, the indirect effect mediated solely by T1 appearance social pressure (SPA) was 0.055 (95% CI [0.028, 0.082], P <0.001). The indirect effect mediated solely by T1 body image was 0.034 (95% CI [0.015, 0.053], P <0.001). Crucially, the chain mediation effect (T0 FNE → T1 SPA → T1 Body Image → T2 SA) was also significant, with an effect size of 0.018 (95% CI [0.007, 0.029], P <0.001). Collectively, the total indirect effects accounted for 72.3% of the total effect, underscoring the dominant role of the proposed mediational pathways.

## Discussion

### Summary of main findings

This prospective study, employing a three-wave longitudinal design over a 6-month period and utilizing cross-lagged panel analysis, delineated the temporal associations among fear of negative evaluation (FNE), appearance social pressure (SPA), body image (BI), and social anxiety (SA) in women with polycystic ovary syndrome (PCOS). Five core findings emerged:

Bidirectional Reinforcement: FNE and SA exhibited significant reciprocal predictive relationships over time, suggesting a self-perpetuating vicious cycle.

Maladaptive Cognitive Cascades: Elevated FNE at one timepoint was prospectively associated with higher SPA and SA, as well as lower BI, at the subsequent timepoint.

Vulnerability Pathways: Higher levels of SPA negatively predicted subsequent BI and positively predicted subsequent SA.

Protective Effect: BI served as a protective factor, negatively predicting future SA symptoms.

Chain Mediation: SPA and BI functioned as significant serial mediators in the pathway linking FNE to SA.

Collectively, these findings address a critical gap in the literature regarding the longitudinal mechanisms of social anxiety in PCOS. Crucially, by establishing temporal precedence through CLPA, this study resolves the ambiguity inherent in previous cross-sectional research regarding the directional relationship between FNE and SA (i.e., whether FNE precedes SA or vice versa). Consequently, these results provide high-level empirical evidence to inform the identification of specific intervention targets for mitigating psychological distress in this clinical population.

### The bidirectional mechanism between fear of negative evaluation and social anxiety

This study provides the first longitudinal evidence of a reciprocal predictive relationship between fear of negative evaluation (FNE) and social anxiety (SA) in women with PCOS, extending prior conclusions limited to cross-sectional designs. From a cognitive-behavioral perspective, FNE acts as a higher-order cognitive vulnerability, prompting individuals to persistently monitor social cues and amplify negative appearance-related feedback, thereby activating anxiety schemas. Conversely, the avoidance behaviors and negative social experiences precipitated by SA reinforce the fear of being scrutinized, creating a self-perpetuating cycle. These findings align with results observed in adolescent and obese populations (28, 29) and corroborate the cross-sectional evidence reported by Jannink et al. in infertile women with PCOS (14).

However, distinct from general population samples, the physical phenotypes characteristic of PCOS serve as potent triggers for FNE. Research by Stener-Victorin et al. (1) indicates that clinical manifestations such as hirsutism and acne can increase perceived negative appearance feedback by 30%–50%, potentially explaining the relatively larger effect sizes for FNE observed in the present study. The PCOS-specific symptom cluster (hirsutism, acne, obesity) provides unique triggering cues for this vicious cycle, suggesting that interventions targeting this population must address the interactive effects of cognitive vulnerabilities and somatic symptoms concurrently.

Mechanistically, this improvement may be attributed to two synergistic factors. First, medical interventions—such as hormonal regulation and dermatological treatments aimed at improving hirsutism and acne—directly reduced sources of physical stigma and decreased external negative feedback regarding appearance (30). Second, the provision of ongoing disease education and clinical support throughout the follow-up period facilitated patients’ gradual acceptance of their pathological features and fostered the development of adaptive coping strategies. This psychological shift likely weakened the pathway linking appearance social pressure to FNE, effectively disrupting the generative mechanism of SA (31).

### The serial mediation of appearance social pressure and body image

While prior cross-sectional studies have identified the independent mediating roles of appearance social pressure (SPA) and body image (BI) separately, the present longitudinal design advances this understanding by confirming their specific sequential chain: fear of negative evaluation → SPA → BI → social anxiety. This sequential pattern aligns with Biderman et al.’s findings in college students (11). However, a key distinction emerges within the PCOS context: unlike general populations where SPA predominantly stems from pervasive aesthetic ideals, SPA in women with PCOS is primarily driven by disease-related physical stigma (e.g., hirsutism, acne, obesity). This disease-specific trigger provides a precise and actionable target for intervention, suggesting that mitigating stigma tied to clinical phenotypes may be more effective than broadly addressing societal beauty standards in this population. The results of this study showed that FNE at T0 significantly predicted the levels of outward social stress, body image, and social anxiety at T1, suggesting that this factor is the core driving force for subsequent psychological symptoms. According to cognitive-behavioral theory, FNE, as a higher-order cognitive susceptibility trait, has a significant spillover effect. High fear keeps individuals in a state of alert to social threats, leading to excessive sensitivity to superficial social cues and amplifying external pressure. Frequent self-examination results in body dissatisfaction and finally activates anxiety cognitive schemas, reflecting the remote role of cognitive factors in determining emotions and behaviors (32, 33).

In addition, external social stress at T0 independently predicted body image and social anxiety at T1, confirming the toxic effect of external environmental factors. When patients with PCOS perceive high external requirements for appearance, the huge gap between their real self and their ideal self serves as a chronic stressor, which directly erodes their self-esteem and body acceptance and prompts them to adopt social avoidance strategies to avoid judgment, leading to impaired body image and social anxiety symptoms (20, 34).

FNE at T1 had a significant predictive effect on body image at T2, and social stress and body image at T1 could independently predict social anxiety at T2, which fully confirmed the continuous and cumulative effect of psychological symptoms. In this process, body image plays a key mediating role. That is, patients’ persistent sense of shame about their own body externalizes their internal pain into tension and avoidance behaviors in social situations, and eventually evolves into social anxiety (35, 36).

Meanwhile, without timely intervention, early cognitive biases and environmental stress will continue to remodel brain pathways through neuroplasticity mechanisms, leading to the deterioration of body image and the fixation of anxiety (37, 38). In addition, the inverse predictive effect of T1 social anxiety on T2 fear of negative evaluation reveals a two-way vicious circle between the two. Patients’ anxiety responses in social interactions can easily induce subtle negative feedback from their partners. This real social experience in turn strengthens patients’ false beliefs about being negatively evaluated, thus further exacerbating the fear and forming a closed loop. Although the predictive effect of T1 social anxiety (SA) on T2 fear of negative evaluation (FNE) was statistically significant, the effect size was relatively modest (β = 0.13). This finding suggests that while a reverse pathway exists, the forward-driving effect of FNE on SA may be more prominent during the early post-diagnostic phase in women with PCOS. The observed small effect size may also be attributable to the relatively short follow-up duration (6 months); longitudinal studies with extended observation periods may reveal larger reciprocal effects over time as maladaptive cognitive-behavioral patterns become more entrenched.

The results of Bootstrap analysis showed that T1 SPA and body image had significant indirect effects in the prediction path from T0 FNE to T2 social anxiety, which confirmed the chain mediating effect of the two in the longitudinal dimension. The results suggest that FNE does not directly induce social anxiety but instead operates through the dual pathways of externalizing perceived stress and internalizing impaired body image, which eventually manifest as social anxiety symptoms (39). These findings synthesize the three major theoretical frameworks introduced in this study. From a sociocultural perspective, individuals with elevated fear of negative evaluation (FNE) are more prone to internalizing rigid societal standards regarding female appearance, thereby perceiving heightened appearance social pressure (SPA) (11). Extending this via the cognitive-behavioral model, such pressure triggers repetitive body surveillance and negative self-evaluation, which subsequently erodes body image (BI) (32). Finally, through the lens of experiential avoidance theory, dissatisfaction with BI motivates individuals to escape or avoid social situations to mitigate emotional distress, ultimately manifesting as social anxiety (SA) (16). To our knowledge, this study is the first to empirically validate the longitudinal viability of this integrated pathway within a PCOS population, thereby broadening the ecological applicability of these established theories to chronic physical health conditions characterized by visible phenotypic alterations.

### Clinical implications

Drawing on our cross-lagged findings, FNE assessed at baseline (T0) emerges as a pivotal precursor to subsequent psychological distress in PCOS. Considering its significant prospective links with heightened appearance pressure, deteriorating body image, and rising social anxiety, routine administration of the BFNE and SAS at initial diagnosis is advisable to flag at-risk patients. While mindfulness body scanning and ACT have proven effective in enhancing body image among general populations (23, 33), their application in PCOS warrants exploration to recalibrate maladaptive cognitive appraisals and curb the internalization of societal appearance standards. Such targeted efforts to restore body image could effectively dismantle the “FNE → appearance pressure → body image → social anxiety” cascade, promoting holistic care. Nevertheless, as an observational study, these strategies require rigorous confirmation through future RCTs.

### Strengths and limitations

This study possesses several notable strengths. First, the adoption of a three-wave longitudinal design coupled with cross-lagged panel analysis (CLPA) established clear temporal precedence among variables, addressing the prevailing reliance on cross-sectional data within PCOS research. Second, we rigorously tested and confirmed measurement invariance across timepoints, ensuring the validity of our longitudinal comparisons. Third, the ample sample size provided sufficient statistical power to detect significant effects.

However, several limitations warrant consideration. First, the single-center design may limit the generalizability of our findings; thus, multi-center studies are needed to validate these results across diverse populations. Second, the exclusive use of self-report questionnaires introduces several potential biases. Social desirability bias may lead participants to underreport distressing symptoms (e.g., social anxiety, body dissatisfaction), while common method variance—though statistically ruled out via Harman’s test—may inflate observed associations among constructs. Third, although we adjusted for treatment status as a covariate, residual confounding may persist due to heterogeneity in treatment adherence and dosage adjustments during follow-up; future studies employing daily diary methods could more precisely capture fluctuations in treatment regimens and symptom severity. Fourth, despite an acceptable attrition rate, the 10.6% loss to follow-up may have led to an overestimation of effects, as dropouts typically exhibit more severe psychological symptoms. Fifth, we did not incorporate potential confounders including social support, illness perceptions, and PCOS-specific somatic symptom metrics (e.g., hirsutism severity, serum androgen levels) into the analytic model. Although BMI was adjusted for as a covariate, residual confounding caused by unmeasured phenotypic features remains a possibility. Future studies that integrate objective clinical markers together with psychological variables will improve the translational precision of the model. Subsequent research should integrate these variables to enhance the explanatory power of the model. Finally, the 6-month follow-up duration precludes observations of long-term psychological adaptation trajectories; extended longitudinal monitoring is necessary to elucidate these developmental patterns.

## Conclusion

Employing rigorous longitudinal design and advanced statistical modeling, this study provides the first empirical evidence of a serial mediation pathway involving appearance social pressure and body image within a PCOS population. By overcoming the inherent limitations of cross-sectional research regarding causal inference, these findings offer a novel theoretical framework and identify specific intervention targets for preventing and mitigating psychological distress in this demographic. Clinically, these results underscore the necessity of routine psychological assessment immediately following PCOS diagnosis. Implementing interventions focused on cognitive restructuring and body image enhancement represents a promising strategy to potentially interrupt the observed cyclical symptom progression; however, definitive causal effects await confirmation in experimental trials.

## Data Availability

The original contributions presented in the study are included in the article/supplementary material. Further inquiries can be directed to the corresponding author.
